# Focal Hyperhidrosis Associated with Recurrent Urinary Tract Infections

**DOI:** 10.1155/2016/3842984

**Published:** 2016-06-09

**Authors:** Dina Ismail, Vidya Madhwapathi, Evmorfia Ladoyanni

**Affiliations:** Department of Dermatology, Dudley Group NHS Foundation Trust, Dudley, West Midlands DY1 2HQ, UK

## Abstract

Hyperhidrosis affects almost 3% of the population and is characterized by sweating that occurs in excess of that needed for normal thermoregulation. It can occur as a primary disease or secondary to underlying clinical conditions. Hyperhidrosis can stem from neurogenic sympathetic over activity involving normal eccrine glands. We report the interesting case of a 75-year-old male patient with a 6-month history of new onset secondary focal hyperhidrosis of buttocks, pelvis, and upper thighs. Each time his symptoms worsened he was found to have culture positive urine samples for* Escherichia coli* (*E. coli*). He underwent urological investigation and was found to have urethral strictures and cystitis. The hyperhidrosis improved each time his urinary tract infection (UTI) was treated with antibiotics and continued to remain stable with a course of prophylactic trimethoprim. We hypothesize that the patient's urethral strictures led to inhibition in voiding which in turn increased the susceptibility to UTIs. Accumulation of urine and increased bladder pressure in turn raised sympathetic nerve discharge leading to excessive sweating. We recommend that a urine dip form part of the routine assessment of patients presenting with new onset focal hyperhidrosis of pelvis, buttocks, and upper thighs. Timely urological referral should be made for all male patients with recurrent UTI. To the authors' knowledge, there have been no other reports of UTI-associated focal hyperhidrosis.

## 1. Introduction

Hyperhidrosis is a condition characterized by sweating that occurs in excess of that needed for normal thermoregulation. It can have significant impact on a sufferer's daily life. It may be localized to specific areas of the body or generalized. It can occur as a primary condition or secondary to underlying disease or medication [1]. Here we report a case of a patient with hyperhidrosis associated with recurrent urinary tract infections (UTIs). To the authors' knowledge, there have been no other reports of UTI-associated focal hyperhidrosis in the literature.

## 2. Case Presentation

A 75-year-old male presented with a 6-month history of new onset hyperhidrosis, affecting his buttocks, pelvis, and upper thighs bilaterally. The excessive sweating was necessitating him to change his underwear several times a day and night. His past medical history included a transient ischaemic attack (TIA), hypercholesterolemia, and reflux oesophagitis. His medications were limited to dipyridamole, lansoprazole, and simvastatin.

On his first presentation to the Dermatology Department, there was minimal hyperhidrosis to appreciate; however, his skin was found to be colder in the areas affected. A urine dip performed was positive for nitrites and was therefore sent for microbiological assessment.* E. coli* was cultured and found to be sensitive to trimethoprim (Table 1). The patient was treated for the UTI accordingly and at his next clinic review six weeks later reported complete resolution of the hyperhidrosis.

Two months later, sweating symptoms recurred in the same distribution. Pus and red blood cells were detected in his urine (Tables 2 and 3). The patient was reviewed again by the dermatology team and a repeat MSU was sent. Once again* E. coli* was cultured from the urine sample (Table 4). He was treated with a seven-day course of nitrofurantoin 50 mg four times daily, according to sensitivities and reported improvement in his hyperhidrosis following this. He was also referred for urological opinion.

A flexible cystoscopy showed urethral strictures and patchy cystitis. This was treated with a ten-day course of trimethoprim 200 mg twice daily followed by four weeks at 100 mg daily. Following treatment with an extended course of trimethoprim, the patient reported a 95% improvement in his hyperhidrosis symptoms.

## 3. Discussion

Hyperhidrosis affects approximately 2.8% of the population. Primary focal hyperhidrosis stems from neurogenic sympathetic over activity involving normal eccrine glands, while secondary hyperhidrosis is caused by medication or disease [2].

Primary or idiopathic hyperhidrosis is limited to specific anatomical sites, for example, axilla, palms, and soles, and presents early in life with no identified underlying disease process. Patients with primary focal hyperhidrosis generally do not sweat during sleep; rather the excessive sweating is often triggered by emotional stress. A genetic predisposition may exist, since 30%–50% of patients have a family history of hyperhidrosis [3]. No histopathological changes in the sweat glands have been observed nor do these patients have increased numbers of or larger sweat glands. [4, 5]. A complex dysfunction of the autonomic nervous system involving both the sympathetic and parasympathetic pathways has been postulated. The eccrine sweat glands responsible for the excessive sweating are innervated by cholinergic fibres from the sympathetic nervous system.

Secondary hyperhidrosis is normally of recent and sudden onset, is generalized, and is due to an underlying medical condition. The pathophysiology is of thermoregulatory nature. Causes include acute and chronic infections, malignancy, respiratory failure, alcohol or drug withdrawal, conditions associated with a high sympathetic discharge, such as cardiovascular shock, and endocrine disorders, such as thyrotoxicosis, diabetes mellitus, hyperpituitarism, and pheochromocytoma [2, 4]. Secondary focal hyperhidrosis is rare and can be a subtle sign of partial nerve damage that can be due to nerve compression (carpal tunnel syndrome, cervical rib), inflammation (tabes dorsalis), or diabetic (peripheral) neuropathy.

Sympathetic nerve fibres are involved in the autonomic regulation of bladder activity and the sympathetic innervation of the bladder originates in the lower thoracic and upper lumbar spinal cord segments (T10-L2). The sympathetic pathway is stimulated when there is an increase in bladder pressure from the accumulation of urine and results in the closure of the internal sphincter [6]. We hypothesize that in our patient the urethral strictures led to increased inhibition in voiding which in turn increased the susceptibility to urinary tract infections and also increased the bladder pressure due to accumulation of urine. The increased bladder pressure in turn raised the sympathetic discharge that possibly led to excessive sweating. It is possible that the same sympathetic nerve fibres responsible for the autonomic regulation of bladder activity may also have played a role in the innervation of the eccrine glands in the girdle area. His past medical history of TIA and hypercholesterolemia suggests the possibility of arterial damage that might be affecting his peripheral nervous system.

## 4. Conclusion

Sweating is a physiological mechanism. However, excessive sweating—hyperhidrosis—can result in substantial individual suffering and has been shown to have a significant adverse impact on daily functioning. Dermatology Life Quality Index scores of patients suffering from hyperhidrosis have been recorded in studies as being high prior to treatment, indicating a reduced quality of life [7]. In patients presenting with new onset focal hyperhidrosis of pelvis, buttocks, and upper thighs, we recommend that a urine dip be performed as part of the routine assessment and recommend that antibiotic treatment be initiated if urine culture is positive. We recommend timely urological referral in all male patients with recurrent urinary tract infections.

## Figures and Tables

**Table 1:** 

Result value	*E. coli*
Organism growth	An isolate
Amoxicillin	R
Augmentin	S
Ciprofloxacin	S
Nitrofurantoin	S
Piperacillin/tazobactam	S
Trimethoprim	S

**Table 2:** 

Result value	22/*μ*L
Pus cells	22/*μ*L
Epithelial cell	2/*μ*L
RBC	19/*μ*L
UF result	No culture required

**Table 3:** 

Result value	17/*μ*L
Pus cells	17/*μ*L
Epithelial cell	2/*μ*L
RBC	14/*μ*L
UF result	No culture required

**Table 4:** 

Result value	*E. coli*
Organism growth	An isolate
Amoxicillin	R
Augmentin	S
Ciprofloxacin	S
Nitrofurantoin	S
Piperacillin/tazobactam	S
Trimethoprim	S
